# Kinematics Calibration and Validation Approach Using Indoor Positioning System for an Omnidirectional Mobile Robot

**DOI:** 10.3390/s22228590

**Published:** 2022-11-08

**Authors:** Alexandru-Tudor Popovici, Constantin-Catalin Dosoftei, Cristina Budaciu

**Affiliations:** 1 Department of Computer Engineering, “Gheorghe Asachi” Technical University of Iasi, 700050 Iasi, Romania; 2 Department of Automatic Control and Applied Informatics, “Gheorghe Asachi” Technical University of Iasi, 700050 Iasi, Romania

**Keywords:** OMR, indoor positioning system, accurate localization, calibration, validation

## Abstract

Monitoring and tracking issues related to autonomous mobile robots are currently intensively debated in order to ensure a more fluent functionality in supply chain management. The interest arises from both theoretical and practical concerns about providing accurate information about the current and past position of systems involved in the logistics chain, based on specialized sensors and Global Positioning System (GPS). The localization demands are more challenging as the need to monitor the autonomous robot’s ongoing activities is more stringent indoors and benefit from accurate motion response, which requires calibration. This practical research study proposes an extended calibration approach for improving Omnidirectional Mobile Robot (OMR) motion response in the context of mechanical build imperfections (misalignment). A precise indoor positioning system is required to obtain accurate data for calculating the calibration parameters and validating the implementation response. An ultrasound-based commercial solution was considered for tracking the OMR, but the practical observed errors of the readily available position solutions requires special processing of the raw acquired measurements. The approach uses a multilateration technique based on the point-to-point distances measured between the mobile ultrasound beacon and a current subset of fixed (reference) beacons, in order to obtain an improved position estimation characterized by a confidence coefficient. Therefore, the proposed method managed to reduce the motion error by up to seven-times. Reference trajectories were generated, and robot motion response accuracy was evaluated using a Robot Operating System (ROS) node developed in Matlab-Simulink that was wireless interconnected with the other ROS nodes hosted on the robot navigation controller.

## 1. Introduction

The high interest in the analysis of the performances of Omnidirectional Mobile Robot (OMR) navigation platforms is increasing in the scientific community [1,2,3], as well as in the industry field for different types of applications, starting from monitoring and mapping of the area of interest towards the transportation, intelligent manufacturing [4], and logistic activities [5,6]. In this context, the OMR vehicle performs movement in any direction under any orientation; therefore, it has great advantages over conventional platforms (i.e., car-like Ackermann steering or differential drive system) in warehouse management, where complex trajectory planning associated with task assignment is a demanding requirement.

Currently, the main attention in the advanced mobile robots research field is paid towards transitioning from automated guided vehicles to autonomous mobile robots. This technological challenge is sustained by developing complex sensors and computational processing power, which offer new navigation capabilities in a dynamic environment with predefined or variable constraints [2,3,7].

The prediction is that over four million logistical robots will be developed and placed in approximately 50 K warehouses by 2025 [8]. The favorable factor for this assumption that will have a big impact on performance in the operational logistic domain is represented by the migration of e-commerce to digital commerce. Many parallel research, both from various academic communities and industrial companies, is closely related to the development of a handling and transport solution in a complex logistic environment, the testing and validation of the experimental OMR localization being an important step in the further development of algorithms [9]. The increased interest in omnidirectional systems is primarily due to their maneuverability in logistics and assembly applications [4,6]. Robot pose estimation is based on odometry, which is defined as a simple positioning method based on the wheel velocity measurements and is usually used in real-time experiments.

Path performance evaluation based on odometry is inconclusive due to the mechanical shortcomings of the experimental OMR, even if the control action is well defined. Furthermore, the main concern related to the errors that appear in the localization of the robot is also justified by the fact that the OMR is intended to work in a dynamic warehouse environment. Even if the improvement of odometry by proper calibration reduces the position errors, the accurate knowledge of the current location can be ensured by an Indoor Positioning System (IPS).

In this research study, the data acquisition from the IPS was exploited in order to monitor the motion response of the OMR and calculate the calibration parameters. Experiments involving typical motion paths for the OMR were performed, so that the longitudinal, lateral, rotational, and composed motions were studied in order to establish the calibration requirements for the kinematic transformations used for commanding the motion of the OMR and monitoring its actual execution.

Tracking performance evaluation scenarios for composed motion reference trajectories having Lissajous curve shapes are comparatively analyzed both from the IPS and estimated odometry position [10]. In the current research, a plurality of sets of data was obtained from multiple experimental scenarios, which were carried out starting from simple orthogonal movements towards to complex trajectories.

The architectural concepts together with the basic description of an omnidirectional embedded real-time robotics system was developed by the authors in the current research project, ROSY-LOGISTIC, and the main results were published in previous articles [11,12,13]. With the development of many open-source technologies, an OMR can be used on almost any scenario due to the availability of software libraries and tools, among which is the Robot Operating System (ROS).

This research tries to capture as comprehensively as possible, in a first phase, the necessary steps for the IPS ROS node integration in the Matlab environment, since these steps are not completely described in the technical literature; thus, for anyone, this can be a time-consuming step. Further in Section 2, the motivation of the paper is more pronounced due to the lack of position accuracy in the experimental path recorded. Section 3 attempts to develop a calibration approach starting from measurements of the motion and odometry errors. Section 4 focuses on the experimental scenarios together with the open-loop motion tracking and performance evaluation. The last section concludes the research and discusses the perspective for the future work.

### 1.1. Related Work on Trajectory Validation for OMRs in Logistic Areas

The accurate knowledge of the current localization and orientation of the mobile robot can benefit largely from a well-calibrated odometry. In outdoor applications, Global Positioning System (GPS) technology offers good positioning accuracy and is used in many automated driving applications. Unfortunately, for indoor localization, GPS technology cannot offer similar reliability because the signals of the satellites lose much strength when penetrating a building.

Low-cost technologies such as WiFi, ZigBee, and Bluetooth Low-Energy (BLE) are radio-frequency-based systems and are widely used in in mobile robots for indoor localization [14,15]. Although they are very popular due to the availability of the hardware, the accuracy of static measurements is in the order of 1 to 4 m [16].

Ultra-Wideband (UWB) has gained interest in indoor positioning of robots, the system relying on the signal travel time for the distance between the mobile robot and static anchors. The mobile robot location is estimated by exploiting approaches such as multilateration and trilateration [17]. In the paper [14], the authors benchmarked the accuracy of different types of indoor positioning systems including the Marvelmind robot. The experimental results demonstrated that, in larger spaces, there are situations where the Marvelmind system cannot not perform measurements because of corrupted packets identified using CRC methods, even if there are not any apparent sources of interference present. This phenomenon became more pronounced for higher data traffic. Obviously, in recent years, considerable progress has been made in terms of positioning systems for mobile robots through the use of the latest sensors and signal processing techniques.

There are several approaches for IPSs with their own advantages and limitations [18]. Usually, a data fusion algorithm is used in the localization system to combine the pose estimates from the two different sources. Several methods exist, such as inertial, visual, laser, LiDAR, or wheel odometry, and any of the methods can be applied in a multisensor fusion algorithm, e.g., visual–inertial odometry [15,19]. Though multisensor fusion approaches are usually used, there is a real benefit in increasing the confidence in the odometry.

The mobile robots’ cost increases significantly with the addition of advanced sensors, but odometry and calibration methods can definitely mitigate the positioning error.

### 1.2. Contributions of the Paper

This work comes as a natural continuation of the previous work [12] of the ongoing research project, which aims to develop transport solutions in complex logistic environments using a fleet of autonomous omnidirectional mobile robots coordinated by a warehouse management system. In our previous work [12,13], we provided baseline experimental results starting from orthogonal movements and continuing with more complex trajectories.

The main contribution of this paper concerns the OMR localization accuracy analysis and proposing a new offline method for the OMR kinematics experimental calibration. In this regard, the effectiveness of the method was verified by comparing the performances between the reference trajectory and the estimated position.

The position provided by Marvelmind was compared to a separately implemented position calculation based on raw point-to-point distances reported by the IPS to evaluate its reliability. The initial OMR trajectory tracking was evaluated, and in addition to the translation velocity correction coefficients [20], the need for translation–rotation cross-talk compensation coefficients was established.

## 2. The OMR’s Hardware and Software Architecture

**Figure 1:**
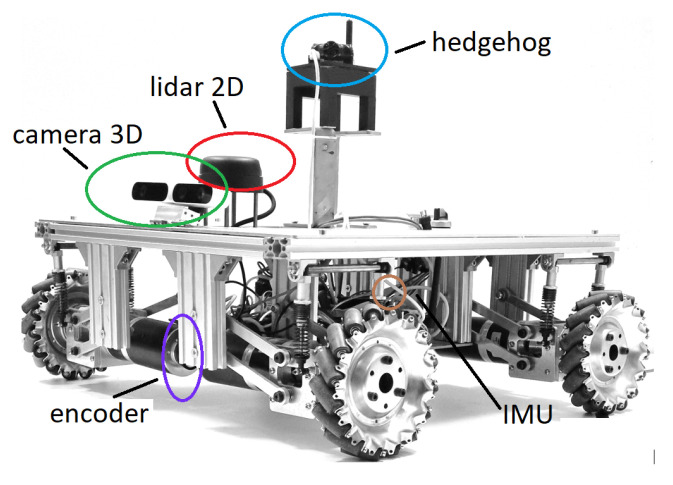
The OMR used in the experiments.

The OMR consists of a chassis that contains the set of mechanical elements including the propulsion system, made up of four motor–planetary gearbox assemblies, coupled to the chassis through eight shock-absorbing suspensions, which ensure contact with the ground, at any time, for the four Mecanum wheels, which have a diameter of 6${}^{\prime \prime }$, as shown in Figure 1. The movement is facilitated by the electrical energy provided with the Li ion batteries, having a nominal DC voltage of 22.2 V. The OMR acts in a working environment to perform different tasks, according to the software component, represented by the implemented control algorithms, which take into account the information received from the perception system. The complexity of the perception system is closely related to the specifics of the operations performed by the robotic platform. The main components with the characteristics of the perception system [21] are presented in Table 1. Last but not least is the control system of the robot.

**Figure 2:**
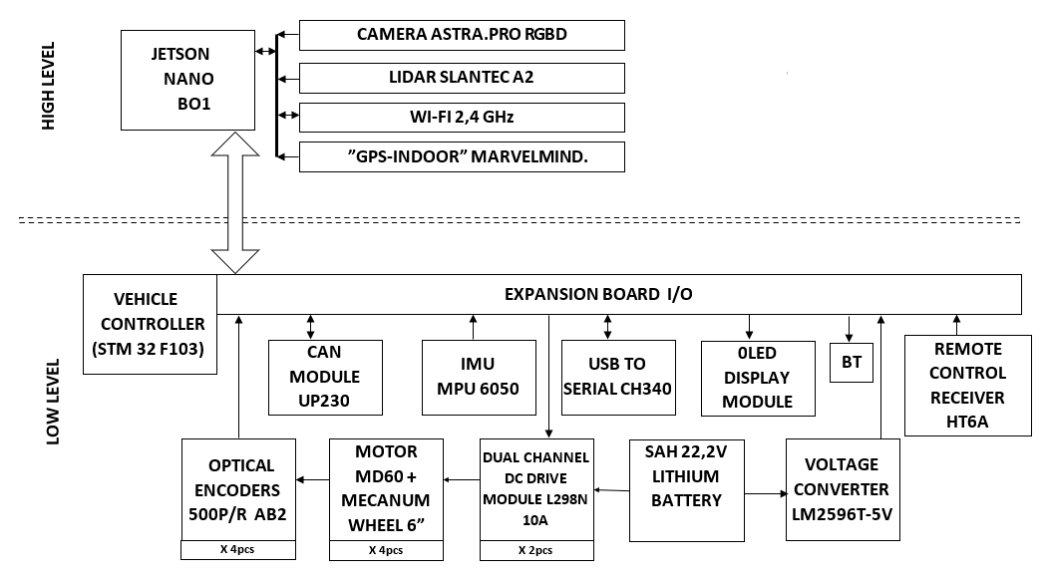
Layered hardware architecture of the OMR.

The driving structure was implemented hierarchically, with a top-down approach, with two controllers: vehicle controller (executive-level) and navigation controller (high-level). The vehicle controller was implemented with an STM32F103RC micro-controller, while the navigation controller was implemented with an NVIDIA Jetson Nano B02 embedded system. The two control systems are directly connected to various components of the robot, as represented in Figure 2, and exchange information through specific control instructions, receiving through the communication protocol both information related to work possibilities and information on the activity and the current state; information analyzed at this decision level allows the robot to further establish the action strategy.

Given the complexity of the software needed in a dynamic environment, a certain degree of computational resources is required. The path-planning algorithms need to be implemented in order to be executed completely on the OMR platform, without the help of external or remote control.

On the low-level controller runs a customized implementation of FreeRTOS. The firmware includes the inverse and direct kinematics models with all specific OMR parameters. As was demonstrated and detailed in [12], the matrix representation of the inverse kinematics is depicted in Equation (1):

(1) $$\left[\begin{array}{c} \omega_{1}\\ \omega_{2}\\ \omega_{3}\\ \omega_{4} \end{array}\right]=J\left[\begin{array}{c} v_{x}\\ v_{y}\\ \Omega \end{array}\right]$$

where ($\omega_{i},i=\bar{1,4}$) represents the angular speed of each wheel, $v_{x}$/$v_{y}$ are the instantaneous longitudinal/lateral velocities component of the OMR, Ω is the rotational speed, and *J* is the inverse kinematic Jacobian matrix of the OMR—expressed in relation to the notational conventions of the OMR elements:

(2) $$J=\frac{1}{R}\left[\begin{array}{ccc} 1 & \;\;\;\;\;1 & -(l_{x}+l_{y})\\ 1 & -1 & -(l_{x}+l_{y})\\ 1 & \;\;\;\;\;1 & \;\;\;(l_{x}+l_{y})\\ 1 & -1 & \;\;\;(l_{x}+l_{y}) \end{array}\right]$$

**Figure 3:**
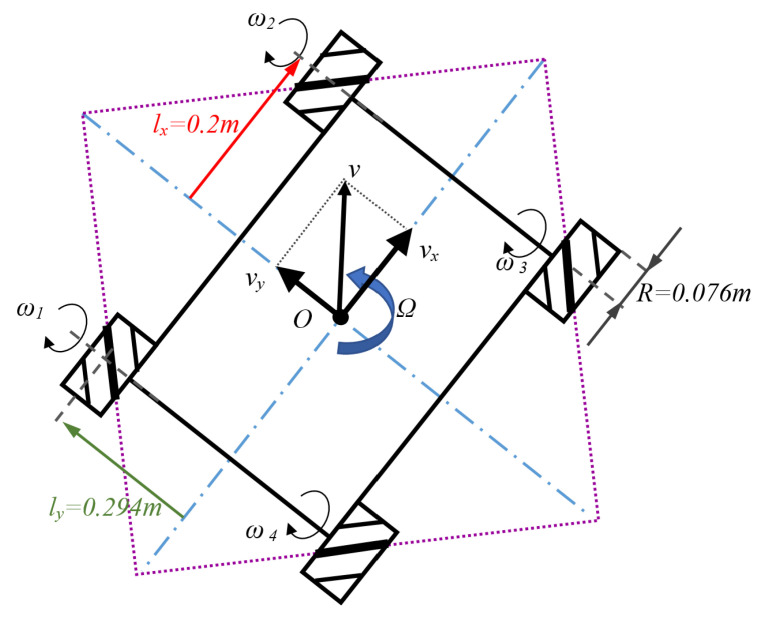
The robot coordinate system and dimensions.

All variables and their numerical values for the experimental platform from Equations (1)–(3) below are highlighted in Figure 3.

The way to determine the lateral/longitudinal and rotation velocities of the OMR starting from each wheel’s velocity is known as forwarding kinematics and is used in odometry calculation—Equation (3):

(3) $$\left[\begin{array}{c} v_{x}\\ v_{y}\\ \Omega \end{array}\right]=\frac{R}{4}\left[\begin{array}{cccc} 1 & 1 & 1 & 1\\ 1 & -1 & 1 & -1\\ \frac{-1}{l_{x}+l_{y}} & \frac{-1}{l_{x}+l_{y}} & \frac{1}{l_{x}+l_{y}} & \frac{1}{l_{x}+l_{y}} \end{array}\right]\left[\begin{array}{c} \omega_{1}\\ \omega_{2}\\ \omega_{3}\\ \omega_{4} \end{array}\right]$$

In an Ubuntu environment with popular programming languages and libraries such as C++, Python, OpenGL, and ROS, the navigation level of the OMR developed on the Jetson Nano, which comes with a Quad-core ARM A57 @ 1.43 GHz and 128-core Maxwell GPU, providing enough resources to cater to the computation for mapping and motion planning.

### 2.1. The Indoor Positioning System—Marvelmind Ultrasound Beacons

The IPS used with the OMR platform was developed by Marvelmind Robotics (Starter Set HW v4.9-IMU-NIA), with a promised accuracy of 20 mm [22], and can be configured to obtain 3D or 2D position solutions. The working principle is based on a network composed of fixed ultrasonic beacons and a mobile beacon, called the hedgehog, installed on the target, which must be localized, in our case, on the OMR. All the beacons are linked by radio interfaces operating in the license-free Industrial, Scientific, and Medical (ISM) band. The mobile beacon on the OMR integrates an Inertial Measurement Unit (IMU) having an accelerometer, a gyroscope, and compass module, which can be used for sensor fusion. The ultrasonic sensor network is completed by a modem having the role of the central controller for the system, which communicates with all the beacons through radio, in the case of the equipment using the ISM 433 MHz band, specific to the European region. Messages with localization information can be received from the hedgehog, but also from the modem, by several communication interfaces (USB Virtual COM Port, I2C, SPI, serial TTL, and others).

There are two operation modes possible for the system, which are called the Inverse Architecture (IA) and Non-Inverse Architecture (NIA). In the IA, the stationary beacons emit ultrasonic signals, while multiple hedgehogs can receive them, the mode being more useful for small areas requiring a minimal number of fixed beacons in order to avoid the reduction of the localization rate. In the NIA, the mode used in the current research, the hedgehogs emit the ultrasonic signals and the stationary beacons receive the propagated wave, and if more hedgehogs are to be monitored in the same area, either Time Division Multiple Access (TDMA) or Multi-Frequency (MF) ultrasonic signals using Frequency Division Multiple Access (FDMA) can be configured for quasi-simultaneous and, respectively, simultaneous tracking. Using TDMA, multiple targets take turns in being localized. Using FDMA, the targets emit simultaneously, but on different ultrasonic frequencies. TDMA implies a reduction of the localization rate, while for FDMA, there is a limited number of frequencies that can be effectively identified using digital filters [23,24] implemented on embedded systems such as the ultrasonic beacons and the coordinating modem.

The location of the OMR is calculated using a proprietary, undisclosed, trilateration algorithm based on the propagation delay, also named Time of Flight (ToF), of the acoustic signal between the hedgehog and up to four nearby stationary beacons of the reference network. The distances between beacons is recommended to be up to 30 m, achieving with just four stationary beacons a coverage area of up to 1000 m${}^{2}$. Larger or more complex areas can be covered using more stationary beacons in the reference network.

The performance of the localization system depends on many aspects, and a proper configuration of the fixed beacon network is essential for achieving the promised accuracy. In real-world scenarios, it can be difficult to achieve the ideal conditions needed for proper operation. Based on the acquired experience, the localization rate decreases on so-called submaps (subareas covered by up to four fixed beacons), where the maximum distance between the beacons is larger. In a 2D configuration for a maximum distance of 5 m between any two beacons, localization rates of around and more than 25 Hz can be achieved. On the other had, 2D localization precision in systems using multilateration is subject to the Horizontal Dilution of Precision (HDoP) [25], which depends on the relative position of the target regarding the available reference beacons. For this reason, even in spaces where there are no obvious issues (propagation path occlusion, multiple/indirect propagation, interference, or other), the accuracy will vary, generally becoming worse when the target is closer to the periphery of the submap in which it is being tracked.

The experimental OMR platform was equipped with the Marvelmind IPS in 2021, and since, then there have been roughly monthly updates of the firmware and software on the producer website, which keeps improving the solution. The latest version used in this study was 7.202 from the beginning of September 2022.

### 2.2. IPS ROS Node for Matlab-Simulink Integration

The Marvelmind company offers as an open-source component, the *marvelmind_nav* Robot Operating System (ROS) package, in order to facilitate the usage of their IPS solutions in industrial and robotics applications. Since it is not yet a standard ROS package, it needs to be manually installed to be used on the OMR. A short summary of the installation steps [26] is presented below:

- Open a command line on the target embedded computer;
- Change the directory to the sources folder (*src*) of the used ROS workspace;
- Make a new directory named *marvelmind_nav* in the *src* folder;
- Download manually the latest ROS package from the Marvelmind repository [22] or use *git clone*;
- Execute *catkin_make -only-pkg-with-depts marvelmind_nav* to build the package;
- Run the node with the command *rosrun marvelmind_nav hedge_rcv_bin /dev/ttyACMx*; in the previous command, the *x* in ttyACMx must be replaced with the device number (usually 1) of the USB virtual serial port that appears in the */dev/* directory when the hedgehog is connected using the USB cable to the embedded computer.

The ROS node named (hedge_rcv_bin) publishes the position data through the topics: *hedge_pos*, *hedge_pos_ang* and *hedge_pos_a*. Marvelmind decided to use custom message types for their published topics, most likely because of the specificity of the IPS application. As a result, until official standard support is offered from ROS and Matlab-Simulink, the following steps will be required for Matlab-Simulink version R2021a in a Windows 10 environment in order to successfully configure the ROS Subscriber blocks for receiving data from the Marvelmind ROS node:

- Check if the message type is available by running in the Matlab Console (MC): *rosmsg list*; if no message name starting with *marvel* appears, than the next steps need to be executed; otherwise, it means that the Simulink model can already subscribe to the position information topics;
- Install CMake 3.15.5 or newer [27];
- Install Microsoft Visual Studio 2017 (VS17), especially the C/C++ development tools and the CMake support (note that, currently, only VS17 is supported for the Matlab ROS toolbox [28]);
- Install Python 2.7 [29] (note that, currently, the Matlab ROS toolbox does not support any other Python version [30]);
- Configure the Python version by executing in the MC (recommended immediately after restarting Matlab): *pyenv(‘Version’,‘2.7’)*;
- Add to the Matlab path the binary installation folder of CMake, by executing in the MC: *addpath(‘C:\Program Files\CMake\bin’)*;
- Set up the Matlab compiler for building the *mex* file type shared libraries with VS17 by executing in the MC (the path depends on the actual MATLAB installation folder): *mex -setup:‘C:\Program Files\MATLAB\R2021a\win64\mexopts\ msvcpp2017.xml’ C++*;
- Copy in Matlab’s current path the folder *msg* from the root of the ROS Marvelmind package [31] that contains *.msg* files;
- Rename the locally copied folder to *marvelmind_ros_messages*;
- Build the needed Matlab *.mex* files for supporting Marvelmind ROS messages by executing in the MC: *rosgenmsg(‘./marvelmind_ros_messages’)*;
- Include the folder containing the support files for custom Marvelmind ROS messages in the Matlab path by executing in the MC: *addpath(‘./marvelmind_ros_messages’)*;
- Save the Matlab path for future restarts by executing in the MC: *savepath*;
- Clear the Matlab workspace classes by executing in the MC: *clear classes*;
- Refresh the Matlab toolbox cache in order to load the new message types by executing in the MC: *rehash toolboxcache*;
- Run the first step again to check that the new message types are now available to Matlab.

Considering that the installation steps have been successfully completed, it should be possible to generate in Simulink the ROS Subscriber blocks to obtain the position of the hedgehog, which can be used to track the OMR for odometry validation. The previous steps are shared in detail because the diversity and the compatibility of the software components needed made it difficult to obtain the complete working solution.

Alternatively, to avoid the cumbersome steps enumerated, the open-source code of the ROS package for Marvelmind can be adapted to generate, in place of the custom Marvelmind message, some standard ROS messages (e.g., point type) that do not need special support, but this path can be challenging also.

### 2.3. Rapid Control Prototyping Using the ROS Node for Matlab-Simulink Integration

**Figure 4:**
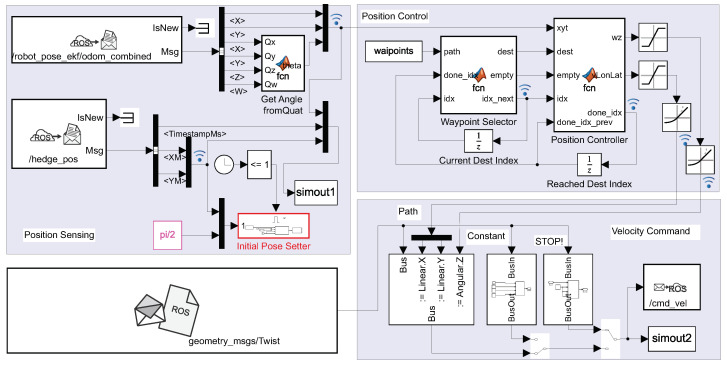
ROS application node implemented in Matlab with odometry initialization from the IPS.

In the initial investigation [13], a simple rapid control prototyping environment was developed using an ROS node designed in Matlab-Simulink, and later, it was developed into the model depicted in Figure 4, where the three main components can be identified: the position acquisition from the odometry and IPS, the position controller used to follow the trajectory described by the waypoint vector, and finally, the ROS blocks used for sending the requested velocity references to the execution layer of the OMR.

The central part of the node is the position controller implemented as a Matlab function block. It was designed to steer the OMR to the prescribed pose received from the waypoint selector block, which keeps track of the current and next target waypoint on the trajectory path. Velocity saturation and acceleration limitation were applied to keep the OMR within nominal parameters and to minimize the risk of damaging collisions.

In the command output stage, manual switches were included so that simple motions and emergency pauses could be requested when the node was executed remotely from a PC, connected through the WiFi network to the rest of ROS nodes running on the OMR. This approach allowed for accelerated testing and improved debugging of the developed node by using Matlab-specific tools such as signal probes and data recordings.

### 2.4. Initial Tracking Results Using the ROS-Matlab Simulink Approach

With a setup similar to the one described in the previous subsection, some initial experiments were carried out using simple trajectories, such as a square shape [13], to investigate the performance of the odometry calculated by the integration of the raw relative speed to the ground based on the direct kinematics described in Equation (3), and the necessity of applying velocity compensations [20] to improve the results became apparent.

**Figure 5:**
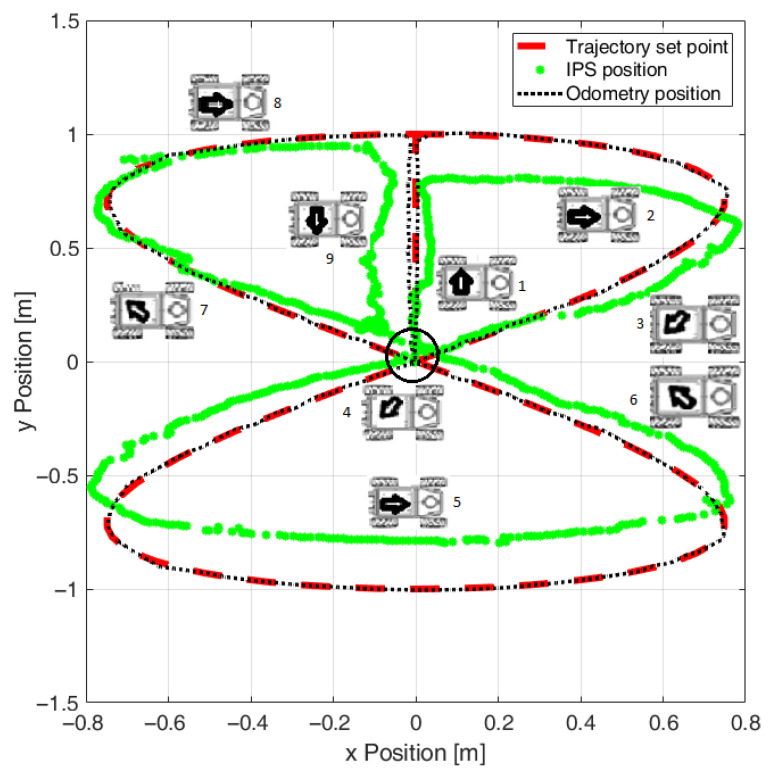
Initial experiments without odometry calibration (the numbered icons indicate the direction and the order of travel on the trajectory segments).

In Figure 5 are illustrated the recorded paths, as measured using the odometry and IPS, against the reference desired path. Since the trajectory controller was configured to work in closed-loop based on the position estimated using odometry, it can be noticed that, although the odometry was tracking the reference, the validation performed with the IPS revealed systematic deviations. Other inconveniences that can be noticed in the path recorded with the IPS, but that were not visible in reality, were the position estimation jitters of the IPS, which appeared especially in certain locations of the experimental area.

At that moment, the quality stream of the Marvelmind IPS localization was not used; however, it became obvious that the configuration of the IPS beacons was not ideal, and in certain areas, some of the stationary beacons missed the direct signal from the hedgehog and received only indirect propagation reflected on nearby walls. This observations motivated a more careful approach to the placement of the IPS stationary beacons, but also the interest in monitoring the reported position quality and attempting an independent trilateration implementation based on the raw beacon-to-beacon measurements, which can be received from the IPS after each localization in order to obtain a quantitative assessment of the position accuracy.

**Figure 6:**
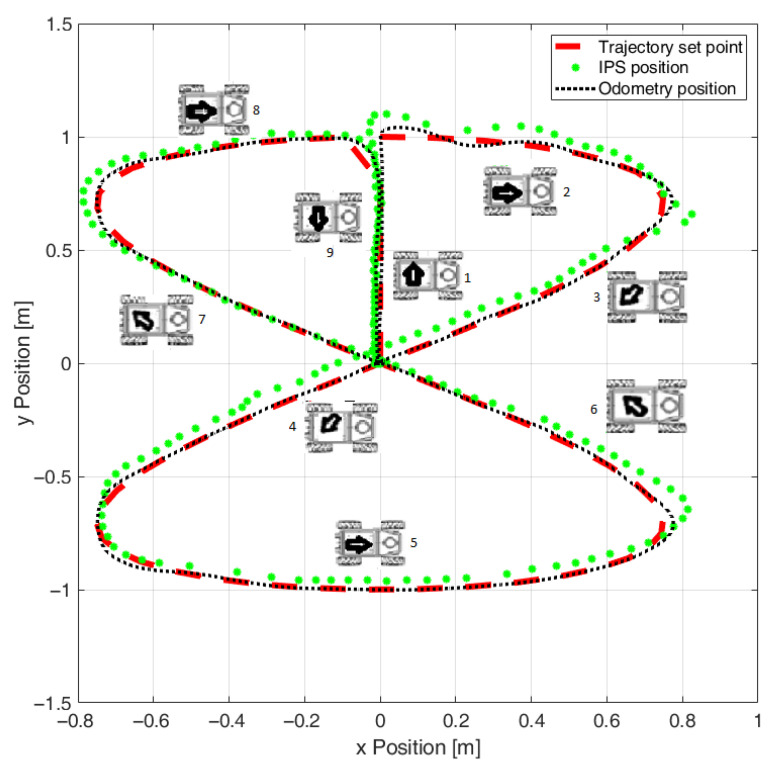
Initial experiments with odometry calibration (the numbered icons indicate the direction and the order of travel on the trajectory segments).

After implementing support for velocity reference compensations [20] at the execution level of the OMR based on the recorded systematic errors, the results from Figure 6 were obtained which showed a significant improvement, especially for the lateral direction. In Figure 6 also, it is visible that the OMR used a higher reference speed since the IPS position samples were more spread out and the overshoot of the trajectory controller was more obvious in the first direction change when starting to follow the actual Lissajous curve shape. By applying the calibration, the significant systematic lateral translation deviation was reduced from about 22 cm to under 5 cm.

Another issue that was identified after the initial calibration was an undesired rotation of the OMR while following the reference track. This aspect is not visible in Figure 6 because the combined odometry provided by the specialized ROS node that also uses the IMU of the OMR managed to keep good track of the azimuth change, and as a result, the trajectory controller could perform an accurate-enough tracking.

## 3. OMR Indoor Navigation Improved by Calibration of the Motion and Odometry

Autonomous navigation benefits from the good accuracy of the motion and odometry. In this process, there are three contradictory requirement concerns: high accuracy, robustness for different conditions, and the application of cost-effective sensors and methods. Good performance can be ensured with high mechanical precision for the OMR, which implies higher costs. Better position-finding sensors can solve the positioning control using a closed-loop approach. This also leads to a higher cost, as well as lower robustness.

In this context, a calibration method designed to compensate through software the undesired operation of the OMR due to mechanical imperfections is of interest since it can improve the base performance for motion- and odometry-based localization, which can only be useful for more advanced techniques such as sensor fusion.

Considering the observed issues regarding the initial results obtained in the previous section, which used a complex control structure made of a path planner based on waypoints and a positioning controller, a simplified open-loop control structure was considered for further analysis and calibration of the odometry. In this way, attention can be concentrated on the performance of the OMR, while other possible sources of errors are limited. As a result, a scripted environment based on Python was developed to apply sequences of motion requests (as relative velocity vectors that the OMR platform can execute) and, at the same time, to record the reported odometry data and the localization information from the Marvelmind IPS for later analysis and calibration information extraction.

**Figure 7:**
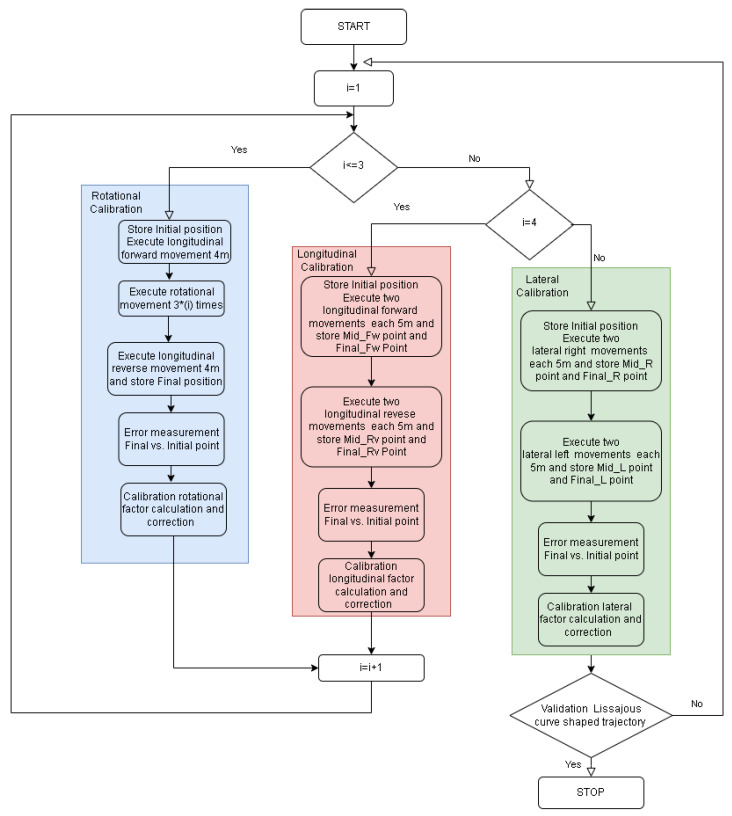
The flowchart of the experimental procedure.

In order to perform the experimental calibration of the OMR kinematics, certain practical challenges need to be taken in consideration and mitigated: mechanical accuracy and play of the OMR components, wheel slippage during aggressive maneuvers, floor quality, suspension response, integration errors, initial position, and orientation estimation. In this context, a practical approach needs to be constructed around simple operations that can be easily reproduced systematically, which motivated a simpler open-loop motion control solution. The entire experimental procedure followed the flowchart from Figure 7.

### 3.1. Open-Loop Motion Response for Orthogonal Movements

In order to obtain good performance for complex movements, it is essential to obtain the desired response for simple orthogonal movements, i.e., pure longitudinal, lateral, or rotational motion, respectively, which are specific to the OMR. The research conducted on the OMR based on four Mecanum wheels showed that the direct usage of the mechanical parameters for solving the kinematic equations led to discrepancies, especially for the lateral and rotational motion. The observed motion errors can be classified into scaling errors (moving/rotating more or less than expected) and cross-talk errors (one orthogonal motion, i.e., lateral, produces another undesired orthogonal motion, i.e., rotational).

### 3.2. Measurements of the Motion and Odometry Errors

Depending on the type of motion error, different methods can be applied for experimental determinations. For translation scaling errors, a laser range finder was considered for measuring the actual motion due to its high accuracy. Alternatively, a well-set-up IPS together with an averaging strategy in specific reference points of a test path can be used to obtain a more complete picture of the actual motion in time. For rotational scaling error and the motion cross-talk errors, the methods were based on localization at relevant points from the reference trajectory using the IPS or the usage of the LiDAR to directly measure the orientation variation relative to some available reference (e.g., a wall). Although the IPS supports a special mode called *paired mobile beacons* to establish the orientation of a target that carries them, the method was not pursued due to its additional setup complexity and also because the starter kit used contained only four stationary beacons; converting one of them to a paired mobile beacon meant a reduction of the fixed beacons used for the multilateration of the position.

An important aspect can be the order in which the motion errors are determined, especially because of the noticed cross-talk from translation to rotation motions. In this context, it is easier to first determine the scaling compensation required for the correct rotation and then attempt the determination for translation and the cross-talk between translation and rotation, which cannot be easily separated. By starting with the determination of the rotation correction factor, then the proper translation–rotation cross-talk factor can be directly determined.

#### 3.2.1. Rotational Movement Error

Depending on the amount of inaccuracy for the kinematics involved in rotation, an iterative practical approach may be required for easier determination. The technique for the iterations was similar, it being necessary in all cases to establish a reference direction, followed by a number of rotations in place, and then, after stopping, a final measurement of the orientation direction. The in-place rotation was executed by requesting the OMR platform to rotate at a fixed rate for the theoretical time needed to complete a specific number of rotations. Considering a firmware implementation on the OMR that limits the acceleration and deceleration to the same value to avoid slippage or over-currents, the previously described requested motion should be executed as expected.

For example, in the first iteration, a single rotation was performed to obtain an initial estimation of the correction factor, which was then applied in the inverse and direct kinematic transformations. In the next iterations, the number of in-place turns was increased to benefit from averaging-out the errors, such as those affecting the measurement of the initial and final orientation.

In the case that the IPS is to be used for determining the rotation correction factors, a method for evaluating the initial and final orientation is necessary if the paired mobile beacons option is not considered, as summarized in the left most (blue) column of Figure 7. The solution is to measure (averaging is recommended to improve accuracy) the initial position at stand-still using the IPS and then perform a longitudinal translation forward at a fixed speed and for a specific time, followed by a stop and a second accurate measurement of the intermediate stand-still position. The initial and first intermediate positions were used to determine the initial orientation by using the arc-tangent trigonometric function in both the IPS and odometry reference system. A number of in-place rotations were executed, followed by a complete stop and then a second orientation determination (based on a second intermediate point and a final point), this time by executing a reverse longitudinal motion. The steps described above were performed pragmatically using a script, which made the measurements and also applied the velocity references to the OMR while calculating in the end the correction factor. Assuming that the translation cross-talk is similar for both forward and reverse longitudinal motion and that the number of rotations is high enough, the correction factor should not be significantly affected by the eventual variation of the cross-talk effect or the imprecision of the IPS. The iterative approach is simple because the total correction factor can be calculated as the product of all the previous correction factors currently determined.

**Figure 8:**
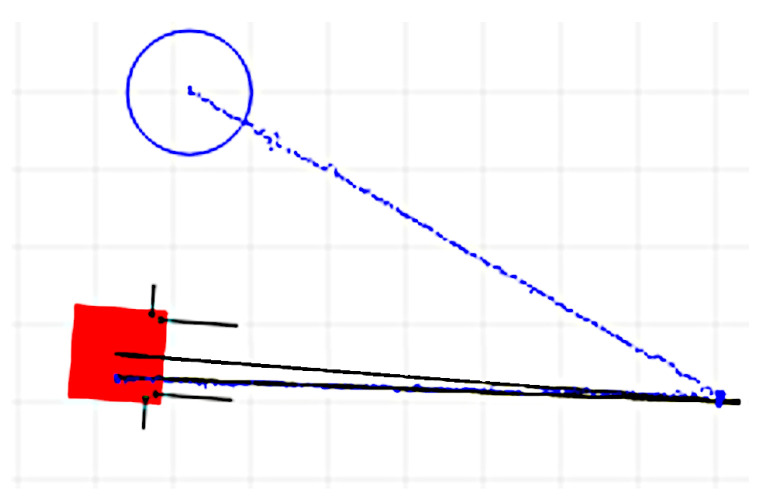
Rotation correction factor determination by using the IPS in the first iteration.

**Figure 9:**
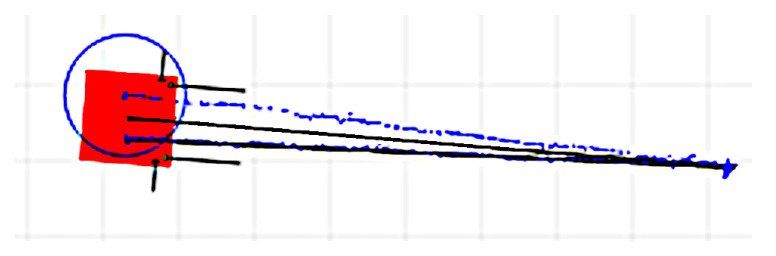
Rotation correction factor determination by using the IPS determination in the second iteration.

To minimize the limitations of the IPS’s accuracy, the longitudinal translations are recommended to be as large as the area in which the IPS is least affected by the HDoP or other issues. In Figure 8 and Figure 9 are presented captures from the web interface used for monitoring the OMR during the rotation correction factor determination after the first and second iterations. The grid spacing was 0.5 m, and the black and blue tracks represent the odometry and the IPS recordings, respectively. The IPS recording is drawn as separate points, useful for visually evaluating the dispersion of the position solutions along the path, certain areas being more affected than others. The light red square represents the current (final) location of the OMR according to the odometry, and the blue circle represents the current (final) IPS reported position. It can be noticed that the odometry position was initialized from the IPS, but no effort was made to align the initial orientation of the odometry and IPS coordinate systems, since this aspect was not relevant for the determination. Additionally, it is visible that the return path of the odometry also had a small deviation compared to the forward path, which can be explained by the approximate method used to execute the rotation by applying a reference rotational speed for a predetermined amount of time. The initial deviation for three complete rotations was determined to about 25.5°, and after calibration, it was reduced to under 3°.

In a similar way, the kinematic rotation correction factor can be determined using LiDAR, in this case without it being necessary to perform the longitudinal motions to identify the initial and final orientation.

#### 3.2.2. Translation Movement Error

As mention in [20], the translation movement errors are relatively easy to determine and then to calculate the velocity correction factors in order to compensate the undesired effects. In the current study, the observed rotation during translation, especially in the lateral direction, can slightly complicate the procedure, requiring a combined approach. In the simplest form, a constant reference speed was applied for a certain amount of time, and the initial and final position provided by the odometry and IPS were used to calculate the correction factor. To simplify the automation of determining the correction factor for translations, it is very useful to also perform the reverse motion so that the experiment is reset for a new determination. This also allows for a second set of data to be extracted.

**Figure 10:**
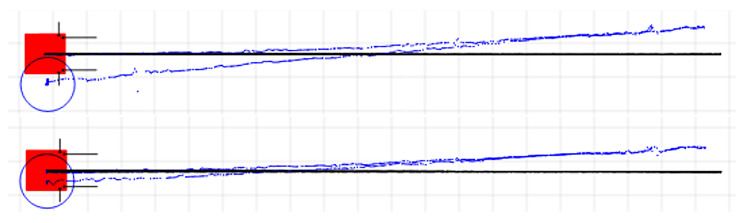
Two experiments for longitudinal translation correction factor determination by the IPS.

**Figure 11:**
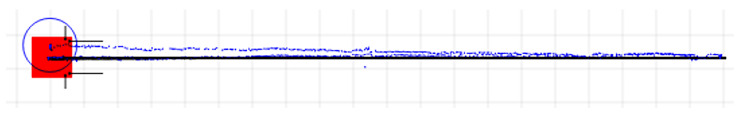
Result after applying the longitudinal translation correction factors obtained by the IPS (translation, forward-translation–rotation, and reverse-translation–rotation correction factors).

In Figure 10 are represented the captures after two experiments for determining the longitudinal translation correction factor using the same representation conventions as in Figure 8 and Figure 9. It can be noticed that the return paths had a slight deviation compared to the forward paths and were not always the same. This can be attributed to the mechanical play of the OMR’s wheel assembly and also to the non-symmetric translation–rotation cross-talk effects for the forward–reverse directions, which is discussed next. In Figure 11 is presented the capture for another iteration of the experiment after the longitudinal correction factors were applied to compensate the kinematics. Besides the longitudinal translation correction factor, two more factors to compensate the rotation during the forward and reverse translations were applied so that the deviations were significantly reduced; more details follow in the next part. The longitudinal translation before calibration was about 25 cm shorter than the 10 m recorded by the odometry, while after calibration, only deviations under 5 cm were obtained.

#### 3.2.3. Undesired Rotational Movement during Simple Translation

**Figure 12:**
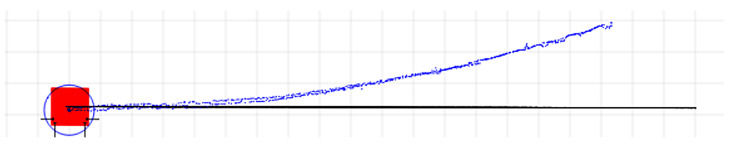
Lateral translation correction factors determination by the IPS (translation, left-translation–rotation, and right-translation–rotation).

Under the assumption that the coupling between the translation velocities and the rotational velocity side effect is linear, the recording of the paths from the IPS correlated with the odometry, as illustrated in Figure 12, can be used to establish the length and the radii of the arcs specific for each type of translation. Since, in the practical experiments, certain differences were identified between the motions in opposite directions (forward–reverse and left–right), it was of interest to determine four separate translation–rotation cross-talk correction factors, in addition to the three kinematic scaling factors described in [20].

By using the IPS, the arc radii can be determined by fitting the recorded path with an arc and then determining its length. The method has the advantage of reducing the impact of eventual IPS measurement errors. The ratio between the odometry displacement and the arc length gives the inverse kinematic translation correction factor. The curvature of the arc, defined as the reciprocal of the arc’s radius, is the translation–rotation cross-talk correction factor used to calculate the compensation rotation velocity to obtain the desired straight motion. In order for the odometry not to measure the injected compensations, the direct kinematics need to be counter-compensated in reverse order with the reciprocal of the correction factors.

As an alternative to fitting, the arc radius can be determined by obtaining three accurate reference points along the track: the start point *S*, a mid-point *M*, and the final point *F*. The disadvantage of the method is that if a mid-stop is executed to obtain an averaged determination based on the IPS, the orientation of the OMR is likely to slightly change and affect the determination. Using the coordinates of the three points, the center *C* and the radius *R* of the circumscribed circle can be determined analytically. Furthermore, using the known vertices of the triangle ▵SCR, the angle ∠SCR can be determined and, then, the exact length of the arc using the radius *R*.

**Figure 13:**
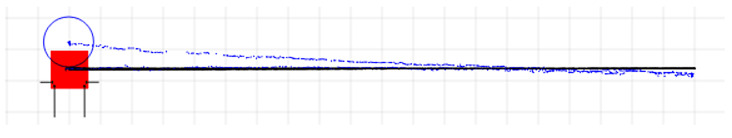
Result after applying the lateral translation and translation–rotation correction factors obtained by the IPS.

As a metric of performance, translation–rotation cross-talk deviations of up to 7 mrad m${}^{-1}$ for longitudinal and 38 mrad m${}^{-1}$ for lateral motion were recorded before calibration, while after calibration, the values decreased to 1.5 mrad m${}^{-1}$ and 5 mrad m${}^{-1}$, respectively, as illustrated in Figure 13.

#### 3.2.4. Experimentally Obtained Kinematic Correction Factors

By applying the previously described approach, the kinematic correction factors listed in Table 2 were obtained for the OMR used in the experiments. It can be noticed that the additional translation–rotation cross-talk compensation factors were similar, but not equal for translations in opposite directions.

## 4. Experimental Setup and Real-Time Validation Subject to Odometry Calibration

In logistic systems and especially in the calibration phase of the OMR’s development, it is beneficial to use an IPS, and it is also essential to measure its accuracy. Considering the unavoidable position solution uncertainties due to the ultrasonic system setup, the manufacturer describes qualitatively the confidence of the latest position solution as a percentage. In the research context of OMR platforms, it can be more useful to obtain a quantitative estimation of the uncertainty that would ensure the assessment of each obtained positioning solution sample.

### 4.1. Evaluation of the IPS and Improvement Attempts

Since there is no open information given about the position solution calculation method for the IPS used, to our knowledge, it became necessary to apply an external/parallel method to validate the offered position solutions, because during our experiments, significant deviations were observed in certain locations.

Among the useful functionalities provided by the API, there is an option for obtaining the raw point-to-point distances between the mobile beacon (named the hedgehog) and each of the up to four fixed beacons in the current localization zone. Thanks to a relatively open approach regarding the parts of the communication protocols used and an extensive API, it is possible to develop external dedicated methods based on trilateration, methods that can also provide a better metric for assessing the accuracy.

**Figure 14:**
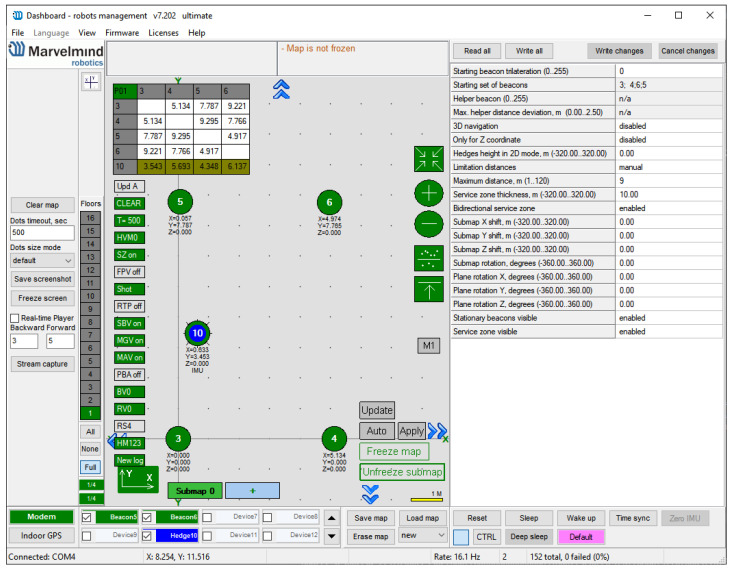
The Marvelmind app dashboard useful in the IPS’s configuration.

The mobile beacon, represented by the blue point in the configuration dashboard in Figure 14, can be configured to provide several useful details for external position solution calculation after each internal position measurement: the position solution calculated internally, the raw point-to-point distances between the hedgehog and up to four fixed beacons involved in the localization, and the latest position quality percentage. The setup and configuration presented in Figure 14 allowed for easier replication of the tracking results.

In addition, the system outputs the position information for all the configured fixed beacons every 10 s, allowing for automatic map updating, which is useful for the external position calculation.

The positioning error is defined as in Equation (4) using the root mean square method over the deviations of the measured point-to-point distances, as reported by the IPS, and the corresponding distances between the estimated position solution and each of the fixed beacons. In Equation (4), $n_{b}$ is the number of beacons (up to four for this IPS) involved in multilateration, $\left(x_{i},y_{i}\right)$ are the coordinates of the beacon *i*, $d_{i}$ is the reported distance between the hedgehog and the beacon *i*, and (x,y) are the coordinates of the estimated position solution.

(4) $$e=\frac{1}{n_{b}^{2}}\sqrt{\sum_{i=1}^{n_{b}}{\left(\sqrt{{\left(x_{i}-x\right)}^{2}+{\left(y_{i}-y\right)}^{2}}-d_{i}\right)}^{2}}$$

Trilateration was performed externally using an iterative implementation that searches for the coordinate pair (x,y), at millimeter resolution, that minimizes Equation (4), which also quantitatively characterizes the uncertainty of the estimated position solution.

**Figure 15:**
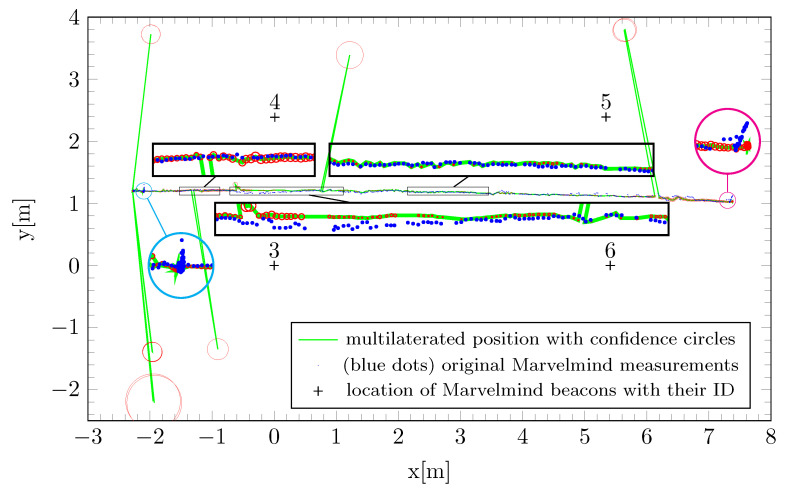
Lateral motion tracking after calibration.

An example of the early attempts to track the calibrated lateral motion using the IPS and the external multilateration technique is depicted in Figure 15, where it can be seen that the unfiltered multilateration solutions were in certain regions strongly affected by indirect propagation of the ultrasound pulses, but the confidence circles helped point out the lack of precision associated with that determination. Furthermore, in some of the zoomed-in regions, especially the center one, there are areas where the multilateration method performed better than the IPS, a fact that was confirmed by both the less-dispersed track, but also by the very tight confidence circles, which indicate a very small RMSE for the solution.

### 4.2. Open-Loop Motion Tracking Performance Evaluation

In order to obtain smoother trajectories, in place of the generic trajectory controller developed using the Matlab rapid prototyping environment, which uses waypoints for navigation, a simplified open-loop trajectory generation was considered.

As a reference path for complex motion test scenario, a Lissajous-curve-shaped trajectory was chosen [20], described in Equation (5), where *S* is a scaling factor (the value *S* = 1 was used in the following tests). φ∈[0,2π] was used to generate the Cartesian coordinate pairs $\left(x_{r},y_{r}\right)$.

(5) $$\left\{\begin{array}{cc} x_{r} & =S\sin\left(2\varphi \right)\\ y_{r} & =S\cos\left(\varphi \right) \end{array}\right.$$

The needed speed vectors that were applied to the OMR in order to obtain the desired motion, without using a position controller, were calculated using the derivative of Equation (5) with respect to φ as in Equation (6).

(6) $$\left\{\begin{array}{cc} v_{x}\left(\varphi \right) & =2S\dot{\varphi }\cos\left(2\varphi \right)\\ v_{y}\left(\varphi \right) & =-S\dot{\varphi }\sin\left(\varphi \right) \end{array}\right.$$

In order to exclude the negative impact of abruptly starting and stopping the OMR when following the Lissajous curve, the track was split into three segments: an acceleration part for which $\ddot{\varphi }=A$ until $\dot{\varphi }$ reached reference speed Ω, then a part executed at $\dot{\varphi }=\Omega$ until $\varphi =2\pi -\frac{\Omega^{2}}{2A}$, the point from which $\ddot{\varphi }$ was set to −A for deceleration during the last part.

**Figure 16:**
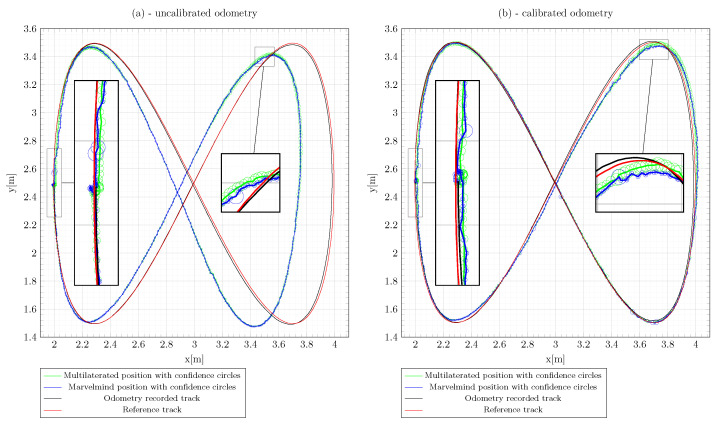
Lissajous-curve-shaped trajectory tracking before (**a**) and after (**b**) kinematics calibration.

**Figure 17:**
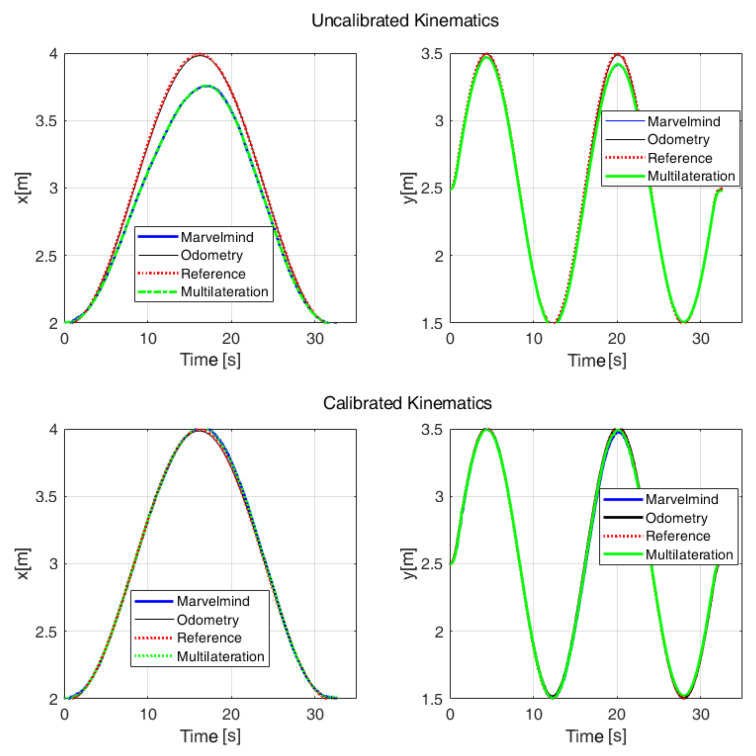
The comparison between the estimated and real position of the OMR on the Lissajous curve trajectory.

In Figure 16 are comparatively illustrated the trajectories using the Marvelmind IPS (blue line), the multilateration method (green line), and the odometry track (black line) with respect to the reference trajectory (red) obtained with the OMR rotated 90° counterclockwise. Lissajous-shaped trajectory are also marked by confidence circles. The maximum deviation of the distances between an estimated and real point was 0.2 m, in the case of uncalibrated odometry. This misalignment is more visible in the time response on the *x* coordinate, as can be observed in Figure 17. To save representation space, the positions of the IPS stationary beacons are not covered in Figure 16, but their identification numbers, types, and positions are listed in Table 3.

In order to evaluate the trajectory accuracy, the root-mean-squared deviation is universally used, based on pose measurements and reference values. The trajectory data used for RMSE evaluation were the Lissajous shape illustrated in Figure 16, where the calculated travel length based on the Euclidean distance was about 10 m. From Table 4, it can be observed that the mean-squared error revealed greater accuracy for the calibrated data compared to the raw values retrieved from the Marvelmind beacons.

## 5. Conclusions

In this study, path performance evaluation was performed starting from orthogonal movements to complex trajectories. The shortcomings of the experimental OMR can be overcome by proper calibration. The initial experimental results revealed that odometry calibration reduced the error propagation, the results being compared with the IPS data. The experiments for calibration were carried out in open-loop with the aim of not interfering with the performance of the navigation controller. The corrections that affected the calibration factors had the role of compensating to a good extent the various constructive imperfections of the robot, such as the wheel assembly error, uncertain wheelbase, and last but not least, the slippage on the running surface.

The OMR was equipped with the Marvelmind IPS for indoor position validation, the acquisition of the data being carried out by using the Matlab environment through ROS application nodes. The evaluation of the IPS was motivated by the lack of open information about the position solution method. Therefore, an external method based on trilateration was proposed in order to provide a better metric, and the root-mean-squared error was used as the performance criterion both in the odometry and the IPS for kinematics evaluation.

Experiments with the OMR showed that the trajectory of the calibrated robot was closer to the ideal trajectory and, thus, could validate the effectiveness of the proposed approach. The experiments were repeated for different speed values of the OMR, and no correlation was observed between the speed and the correction parameters, under the conditions in which the acceleration was kept within the limits, in which it did not produce slips in the starting and stopping modes.

To overcome the drawback of different sensors for indoor positioning and methods, the future approach is the fusion method for the different sensors of the OMR (LiDAR, IMU, stereo camera, encoders, and IPS) to achieve highly accurate, precise position and navigation data.

The whole thread of the work was from the perspective of a systematic approach, the experimental design being a well-defined research methodology, useful to have greater trust in odometry.

The future research direction involves GPS fused with an IMU, the sensor being slightly attached to the GPS in order to predict and update the vehicle position even in the event of GPS signal loss.

## Figures and Tables

**Table 1:** Components of the perception system.

Component Model	Specifications
RP LIDAR A2M8 360	2D LiDAR, resolution: 0.5 mm–1.5 m at a maximum range: 12 m
ASTRA PRO Depth	3D camera stereo, distance: 0.6 m–8 m 1280 × 720 @30 fps
MPU6050	IMU 3-axis accelerometer and gyroscope module
EI 500P/R	Quadrature Optical Encoder 500 ppr
HW v4.9-IMU-NIA	MARVELMIND “GPS” indoor system with beacons

**Table 2:** Correction factors.

Type	Factor	Unit of Measure
Longitudinal velocity	1.02	scaling—no unit
Lateral velocity	1.13	scaling—no unit
Rotational velocity	1.0236	scaling—no unit
Longitudinal-forward velocity to rotational	0.0072	rad m${}^{-1}$
Longitudinal-reverse velocity to rotational	0.00009	rad m${}^{-1}$
Lateral-left velocity to rotational	0.0389	rad m${}^{-1}$
Lateral-right velocity to rotational	0.0277	rad m${}^{-1}$

**Table 3:** Map coordinates of the IPS beacons used for 2D localization.

Beacon ID	Beacon Type	X Position (m)	Y Position (m)	Z Position (m)
3	fixed	0.000	0.000	0.400
4	fixed	5.767	0.000	0.400
5	fixed	0.485	4.809	0.400
6	fixed	5.445	4.813	0.400
10	mobile	-	-	0.400

**Table 4:** Root-mean-squared trajectory tracking deviation.

RMSE	Uncalibrated Kinematics		Calibrated Kinematics	
	Odometry	IPS	Odometry	IPS
Position (m)	83 × 10${}^{-4}$	1119 × 10${}^{-4}$	85 × 10${}^{-4}$	171 × 10${}^{-4}$
*x* (m)	89 × 10${}^{-4}$	1518 × 10${}^{-4}$	86 × 10${}^{-4}$	168 × 10${}^{-4}$
*y* (m)	76 × 10${}^{-4}$	445 × 10${}^{-4}$	83 × 10${}^{-4}$	173 × 10${}^{-4}$

## Data Availability

The data are available upon request from the authors.

## Abbreviations

API	Application Programming Interface
BTSPP	Bluetooth™ Serial Port Profile
CAN	Controller Area Network
FDMA	Frequency Division Multiple Access
GPS	Global Positioning System
HDoP	Horizontal Dilution of Precision
IA	Inverse Architecture
IMU	Inertial Measurement Unit
IPS	Indoor Positioning System
ISM	Industrial, Scientific, and Medical radio band
LiDAR	Light-based Detection and Ranging system
MC	Matlab Console
MF	Multi-Frequency
NiA	Non-Inverse Architecture
o-LED	organic Light-Emitting Diode
OMR	Omnidirectional Mobile Robot
OS	Operating System
RC	Radio remote-Controlled
RMSE	Root-Mean-Squared Error
ROS	Robot Operating System
FreeRTOS™	Free Real-Time Operating System
TDMA	Time Division Multiple Access
ToF	Time of Flight
USB	Universal Serial Bus
VCP	Virtual Serial COM Port
VS17	Microsoft Visual Studio 2017
